# Rheumatoid arthritis and risk for Alzheimer’s disease: a systematic review and meta-analysis and a Mendelian Randomization study

**DOI:** 10.1038/s41598-017-13168-8

**Published:** 2017-10-09

**Authors:** Stefania Policicchio, Aminah Noor Ahmad, John Francis Powell, Petroula Proitsi

**Affiliations:** 1 King’s College London, Institute of Psychiatry, Psychology and Neuroscience, London, UK; 2 University of Exeter Medical School, Royal Devon & Exeter NHS Foundation Trust, RILD Medical Research-Complex Disease Epigenetics Group, Exeter, UK; 3 GKT School of Medical Education, London, UK

## Abstract

Rheumatoid arthritis (RA) patients have been observed to be at a lower risk of developing Alzheimer’s Disease (AD). Clinical trials have showed no relationship between nonsteroidal anti-inflammatory drug (NSAID) use and AD. The aim of this study was to establish if there is a causal link between RA and AD. A systematic literature review on RA incidence and its link to AD was carried out according to the PRISMA guidelines. Eight case-control and two population-based studies were included in a random effects meta-analysis. The causal relationship between RA and AD was assessed using Mendelian Randomization (MR), using summary data from the largest RA and AD Genome Wide Association (GWA) and meta-analysis studies to date using a score of 62 RA risk SNPs (p < 5 * 10^−8^) as instrumental variable (IV). Meta-analysis of the literature showed that RA was associated with lower AD incidence (OR = 0.600, 95% CI 0.46–0.77, p = 1.03 * 10^−4^). On the contrary, MR analysis did not show any evidence of a causal association between RA and AD (OR = 1.012, 95% CI 0.98–1.04). Although there is epidemiological evidence for an association of RA with lower AD incidence, this association does not appear to be causal. Possible explanations for this discrepancy could include influence from confounding factors such as use of RA medication, selection bias and differential RA diagnosis.

## Introduction

Characterized by the development of amyloid plaques and neurofibrillary tangles in the brain, Alzheimer’s Disease (AD) is the most common type of dementia. It impairs an individual’s ability to carry out basic tasks such as bathing and eating. Cognitive function declines, and patients in late stages may be unable to recognize loved ones and become dependent on constant care. Ultimately, AD is fatal^1^.

Information transfer at the synaptic junctions of the brain is compromised in AD patients due to the accumulation of extracellular beta amyloid, which also contributes to neuronal cell death. Inside the neurons, increased levels of tau, a protein that stabilizes microtubules, forms neurofibrillary tangles that block transport of nutrition and necessary molecules throughout the cell. This process also contributes to neuronal cell death^1^.

Pharmacological treatments of AD are limited, with current therapies improving symptoms only transiently. These include cholinesterase inhibitors such as donepezil and rivastigmine, in mild to moderate cases^2^, and memantadine, an NMDA receptor antagonist, in moderate to severe cases. Disease modifying treatments that affect the underlying AD pathogenesis are, as yet, unavailable. The optimal time to administer such treatments may be during the early stages of the pathophysiological process of the disease, before it is clinically apparent^3^.

Neuroinflammation has been linked to AD pathology, although the lack of pain fibers in the brain means the classic signs of inflammation, such as pain and swelling, are absent^4^. Astrocytes and microglia cluster at sites of amyloid beta deposits. Microglial expression of C/EBPβ, a protein that regulates pro-inflammatory molecules such as interleukin 6 (IL-6), interleukin 8 (IL-8), granulocyte colony-stimulating factor, tumour necrosis factor alpha (TNF-α), complement C3 and C-reactive protein (CRP), is significantly increased in the AD cortex compared to the non AD cortex^4^. Additionally, astrocytes are thought to secrete many of the same pro-inflammatory molecules, although less is known about their role in inflammation in AD^4^. A study in which lipopolysaccharide (LPS) was used to produce chronic neuroinflammation in rats reproduced some of the features of AD, such as working memory deficit, elevated levels of beta amyloid precursor protein, and temporal lobe pathology associated with cell loss^5^.

Neuroinflammation responses can be induced by both CNS-intrinsic factors and systemic influences^6^ and a number of conditions such as diabetes^7^, obesity^8^, atherosclerosis^9^, depression^10^, psoriasis^11^ and cardiovascular disease^12^, which have been proposed as risk factors for the development of AD are associated with chronic inflammation^13^.

Rheumatoid arthritis (RA) is an autoimmune disease characterized by synovial inflammation, destruction of bone and cartilage, and autoantibody production, causing progressive disability^14^. It also has systemic features, and frequently occurs with a variety of co-morbidities, some of which have significant effects on its outcome and have been linked to its inflammatory pathogenesis such as cardiovascular disease^15–17^, diabetes mellitus^18,19^ and depression^20,21^.

Paradoxically, RA, itself an inflammatory condition, which is also associated with risk factors for AD, is often considered a negative risk factor for the development of AD^22^. Although, overall the incidence of RA in AD patients has been found to be reduced^23–30^ compared to healthy controls, contradictory results have been reported, with some studies reporting an increased incidence of RA in AD patients^31,32^. However, the majority of these studies have used a small number of AD and RA patients and there is a lack of more recent, large-scale studies.

While it has been assumed that the protective effect of RA is due to the Nonsteroidal anti-inflammatory drugs (NSAIDs) that are used to treat it^33^, a recent randomized controlled trial (RCT), the Alzheimer’s Disease Anti-Inflammatory Prevention Trial (ADAPT), has shown that treatment with naproxen and celecoxib did not reduce AD incidence at 7 year follow up^34^. Additionally, a trial of the TNF-α inhibitor etanercept, another drug used to treat RA, caused no significant changes in cognition, behaviour or global function when recently trialled in AD patients^35^. A systematic review and meta-analysis published by McGeer *et al*. in 1996^22^ investigated arthritis itself, in addition to NSAID use, as a protective factor against AD. This study found that arthritis itself was associated with reduced AD incidence. The overall odds ratio, using arthritis as an AD risk factor, was 0.56^22^.

Here, we attempted to dissect the nature of the association between RA and AD by first employing a systematic literature review and an up-to-date meta-analysis of published epidemiological studies. As a fundamental limitation of observational data is that causation cannot automatically be inferred from an association between an exposure and a disease, as the association could be due to unobserved confounding or reverse causation^36^, Mendelian Randomization (MR) was then employed to formally investigate the causal association of RA with AD.

MR is an established tool for probing questions of causality in order to characterize the aetiology of disease^36,37^. It exploits the fact that genotypes are randomly assorted at meiosis, and are thus independent of conventional confounding factors and the disease process. Therefore, genetic variants associated with intermediate traits can be used to provide an unconfounded estimate of the causal association between the intermediate trait and disease outcome, unaffected by reverse causality. This is akin to a “genetically randomized trial” and the genetic variants are known as Instrumental Variants (IVs). The following three assumptions are necessary for a genetic variant to be a valid IV: 1. the variant is predictive of the exposure; 2. the variant is independent of any confounding factors of the exposure—outcome association; 3. the variant is conditionally independent of the outcome given the exposure and the confounding factors. However, MR studies may be potentially confounded by pleiotropy (association of a genetic variant with more than one trait). Whereas vertical pleiotropy (where a genetic variant affects more than one point in the same causal pathway) does not necessarily breach the assumptions of MR, horizontal pleiotropy, where the variant affects more than one independent causal pathways, can lead to spurious conclusions about causality^38^. Nevertheless when multiple genetic variants are used as IVs there is a higher chance that pleiotropic effects might become balanced and causal inference is possible. Additionally, new methods that can give valid estimates in the presence of pleiotropy have been recently developed. We therefore also used, in addition to multi-SNP Inverse-Variance Weighted (IVW) MR (conventional MR), two recently developed methods; Egger-MR^39^ regression to test for unbalanced pleiotropy and provide a causal estimate of exposure on outcome in its presence, and weighted median MR^40^ which can give valid estimates even in the presence of horizontal pleiotropy if at least 50% of the weight comes from valid IVs. In the absence of horizontal pleiotropy, all three tests should be consistent.

The suggestion that it is RA itself that is protective, if found to be true, could contradict the current theory that AD pathogenesis is worsened by inflammatory processes in the periphery.

## Results

### Studies investigating Rheumatoid Arthritis as a Risk Factor for Alzheimer’s Disease

Ten studies were included in the review and meta-analysis of the literature and provided data on 6346 study subjects. Publication dates ranged from 1983–2002, and there were large variations in both study design and sample size. 8 of the included studies were case control, while the remaining 2 were population-based studies (Supplementary Figure 1).

The characteristics of the 10 included studies that investigated RA as a risk factor for AD, and are therefore included in the meta-analysis, are summarised in Table 1. Studies that included “arthritis” as a risk factor without investigating RA specifically are indicated with an^1^ in the results table. Regarding the Lindsay *et al*. paper^29^, the Odds Ratio omitting decedents was used, as diagnosis of the type of dementia in these participants was not carried out, and may have included other dementia subtypes such as vascular dementia.

**Table 1:** Studies included in the meta-analysis.

Case Control Studies							
Study	Year	Incidence of RA (AD)	Incidence of RA (controls)	Odds Ratio	95% CI	Details of Study Population	NOS Total Score
Heyman *et al*.^32^	1983	16/40 (40%)	29/80 (36%)	1.17^1^	0.54–2.56	Cases: 12 males and 28 females, all Caucasian. Mean age 60.8 at time of study admission.	6/9 (medium)
						Controls: Matched for age, sex and race	
						Study Location: Duke University Medical School, North Carolina, USA	
French *et al*.^26^	1985	?/78	?/76	0.62^1^	0.29–1.29	Cases: 78 Caucasian males.	7/9 (medium)
						Controls: Matched for age, sex and race. Separate neighbourhood and hospital control groups.	
						Study Location: Veterans Administration Medical Centre, Minnesota, USA	
Jenkinson *et al*.^27^	1989	2/96 (2%)	12/92 (13%)	0.14	0.03–0.65*	192 inpatients of a geriatric unit. All 65 years or older.	5/9 (low)
						Study Location: Hackney Hospital, London, UK	
Graves *et al*.^31^	1990	8/130 (6.2%)	5/130 (3.8%)	1.18	0.35–3.91	Cases: 130 patients from a geriatric and family clinic	6/9 (medium)
						Controls: Matched where possible for age, sex, education, socioeconomic and marital status.	
						Study Location: Washington, USA	
Broe *et al*.^24^	1990	92/170 (54%)	115/170 (68%)	0.56^1^	0.36–0.87	Cases: Clinically diagnosed AD patients aged 52–96 years from a dementia clinic	7/9 (medium)
						Controls: Matched for age and sex	
						Study Location: Sydney, Australia	
Li *et al*.^28^	1992	4/70 (5.7%)	35/140 (25%)	0.16^1^	0.05–0.51	Cases: Clinically diagnosed AD patients from psychiatric hospitals and neurology clinics	6/9 (medium)
						Controls: Age and sex matched neighbourhood controls.	
						Study Location: Beijing, China	
Can. Health^25^	1994	104/201 (52%)	280/468 (60%)	0.54^1^	0.36–0.81	Cases: AD subjects age 65 + from both institutions and the community	7/9 (medium)
						Controls: Neighbourhood controls	
						Study location: 36 cities across Canada	
Brietner *et al*.^23^	1994	7/50 (14%)	11/50 (22%)	0.64^1^	0.22–1.77	Co-twin control study on twins with AD onset separated by 3 or more years. Subjects found using the US National Academy of Sciences Research Council Registry	5/9 (low)
						Study Location: USA	
**Population-based Studies**							
**Study**	**Year**	**Incidence of RA in AD patients**	**Incidence of RA in controls**	**Odds Ratio**	**95% CI**	**Details of Study Population**	**NOS Total Score**
Tyas *et al*.^30^	2001	19/35(54%)	360/651 (55%)	0.81^1^	0.39–1.68	Longitudinal study based on randomly selected subjects of 65 or older who were initially cognitively intact.	9/9 (high)
						Study Location: Manitoba: Canada	
Lindsay *et al*.^29^	2002	90/167 (54%)	2013/3452 (58%)	0.61^1^	0.43–0.87	Longitudinal study based on randomly selected subjects 65 or older from all 10 Canadian provinces	9/9 (high)
						Study Location: Nationwide across Canada	

The study characteristics of the 8 case control and 2 population-based studies included in the meta-analysis. 95% confidence intervals (CIs) and brief descriptions of the study populations, as well as the total NOS scale scores are included.

*Calculated based on raw data. Data was assumed to be unadjusted.

^1^Study investigated ‘arthritis’ with no subtype for RA specifically.

8 studies showed RA as a negative risk factor AD, 6 of which were case control and 2, population-based^23,24,26–30,41^, of which 5 showed a significant risk reduction^24,27–29,41^. The remaining 2 showed that RA increased the risk of AD development^31,32^ (Table 1).

### Meta-Analysis of Studies

**Figure 1:**
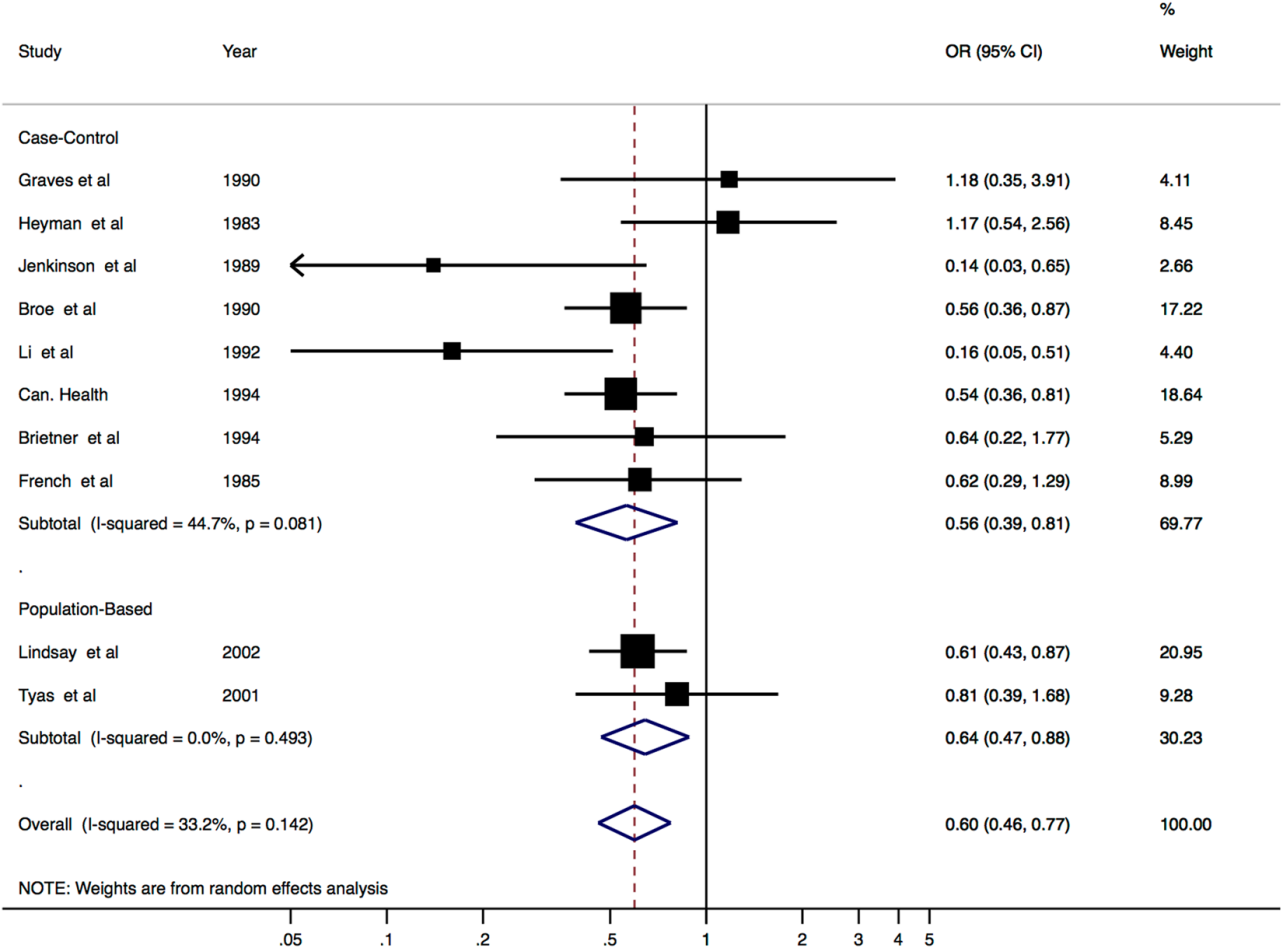
Literature Review meta-analysis results. The results of the meta-analysis, showing separate ORs for the case control and population-based studies, and then an overall OR, 95% CIs, study weight and heterogeneity are also shown.

The results of the meta-analysis of the above 10 studies, including the weight assigned to each individual study, are shown below in Fig. 1. We performed a meta-analysis both separately for case-control and population studies and combining the two.

The meta-analysis results showed that, overall, RA did significantly reduce the risk of developing AD (OR = 0.60, 95% CI 0.46–0.77, p = 1.03 * 10^−4^). This is supported by both the analysis of the case control studies (OR = 0.563, 95% CI 0.39–0.812, p = 0.002) and the population-based studies (OR = 0.644, 95% CI 0.47–0.88, p = 0.006).

### Assessment of Study Heterogeneity and evaluation of bias

Heterogeneity (I^2^) was low in the population-based studies, where it was estimated to be responsible for 0% of the variability. In the case control studies, it was responsible for an estimated 44.7% of variability. The overall I^2^ value was 33.2%.

Meta-regression analysis investigating study year (p = 0.894), study type (p = 0.651), quality of study (p = 0.661), age of participants (p = 0.830), percentage of RA in controls (p = 0.986) and whether RA specifically or arthritis was investigated (p = 0.840) indicated that that there was no significant change at the (log)OR of the outcome per unit increase of these covariates. For age, studies were assessed on whether or not the study included participants < 65.

Potential publication bias was explored graphically, producing the funnel plot shown in Additional File 2, which indicated a slight presence of asymmetry. Both Begg’s and Egger’s tests were therefore carried out to check for publication bias, and both showed that there was no evidence of bias (p = 0.929 and p = 0.668 respectively). Omitting the included studies one at a time indicated that no individual studies had any significant effect on the overall results.

The results of the meta-analysis therefore indicated a consistent protective association of RA and AD.

### Mendelian Randomization Results

**Figure 2:**
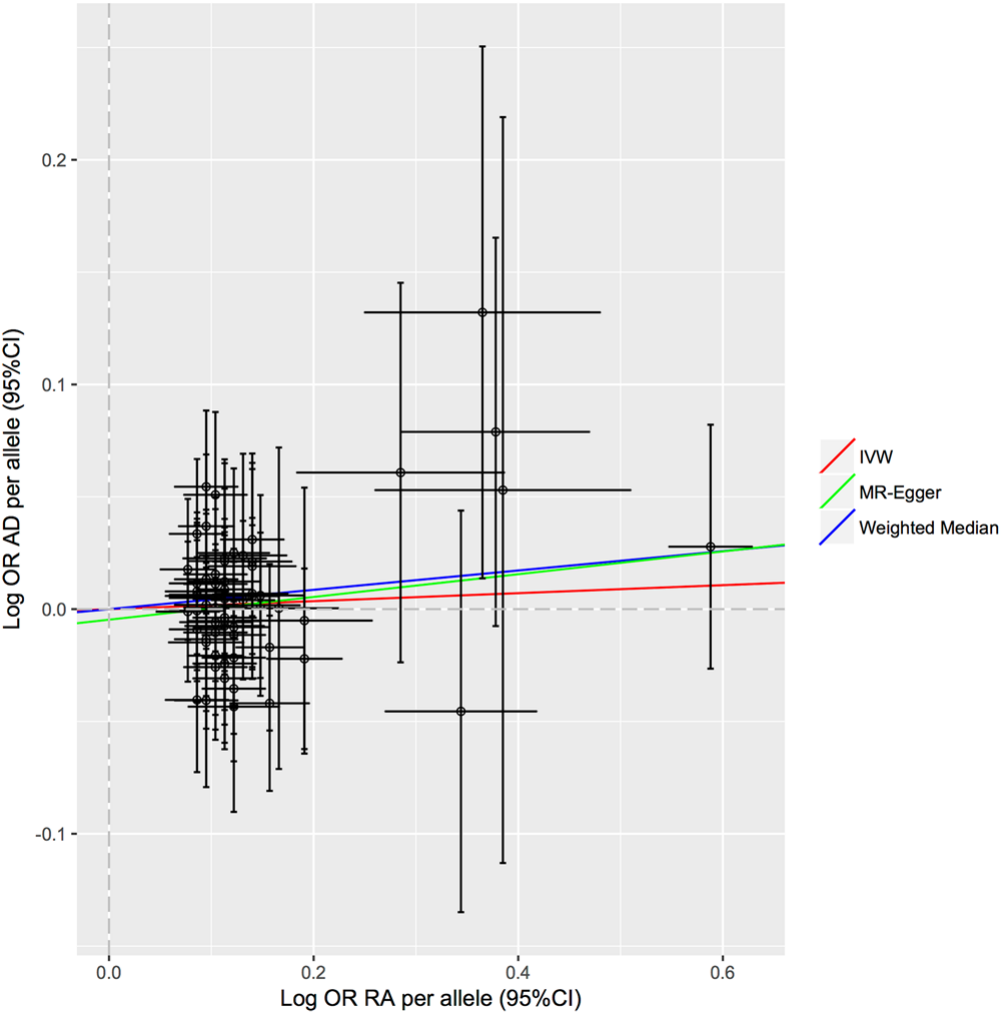
Bidirectional plot. Association of individual SNPs with RA and AD risk. The slopes each line represent the causal association for each method.

Although the overall results of the literature meta-analysis indicate that RA was associated with lower AD risk (OR = 0.60, 95% CI 0.46–0.77; p = 1.03 * 10^−4^), the summary causal estimate from conventional IVW MR showed no evidence to support a causal inverse association between RA and AD (OR = 1.018, 95% CI 0.98–1.06) (Table 2, Fig. 2). Similar results were observed when we used the Median Weighted (OR = 1.04, 95% CI 0.98–1.11), and the MR-Egger (OR = 1.05, 95%CI 0.94–1.17) estimators, and the MR-Egger regression suggested little evidence for unbalanced pleiotropy in the genetic instrument (intercept p = 0.51) (Table 2).

**Table 2:** Results of the Mendelian Randomization analyses using different estimators.

Analysis Method	OR	95% CI	P-value	P-value (intercept)
Inverse-Variance Weighted (IVW)	1.018	0.98–1.06	0.354	
Weighted Median	1.044	0.98–1.12	0.200	
MR-Egger regression	1.052	0.94–1.17	0.363	0.513

The intercept P-value can be interpreted as an estimate of the average (horizontal) pleiotropic effect across the genetic variants.

Finally, the “leave one out” results show that by omitting the included 62 SNPs one at a time, no individual genetic variants seem to have any significant effect on the overall results and after excluding 20% of the SNPs at a time (100000 times) 0.009% of the sensitivity coefficients layed outside the MR 95% CI highlighting that these results are not sensitive to SNP selection.

The MR analysis results therefore do not support a causal inverse association between RA and AD.

## Discussion

**Figure 3:**
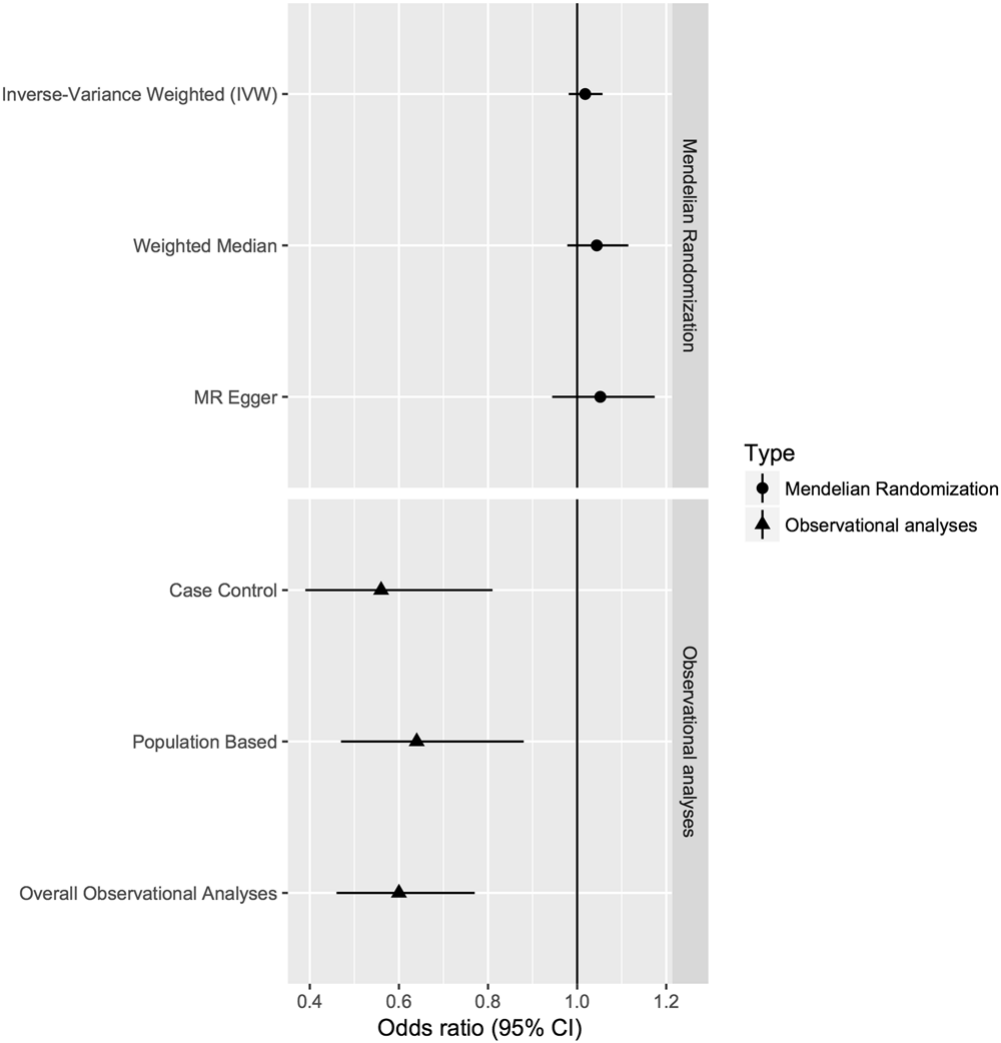
Meta-analyses and Mendelian Randomization analysis results for the association of RA with AD using different methods.

We conducted a comprehensive review and up to date meta-analysis of studies investigating the association of RA with AD, followed by a MR analysis. The findings of these two types of analyses are contradictory (Fig. 3).

The overall results of the meta-analysis indicated that RA is associated with lower AD risk with an overall OR estimate of 0.60 (95% CI 0.46–0.77, p = 1.03 * 10^−4^), a finding very similar to one proposed by McGeer *et al*. in 1996^22^ in the earlier meta-analysis (OR = 0.556, 95% CI 0.44–0.70). This analysis therefore supports their work even with the inclusion of more recent population-based studies.

MR is a powerful tool to dissect causal relationships between an exposure and an outcome as it minimizes residual confounding by lifestyle factors, underlying ill-health, medication and selection bias present in observational studies. We performed three MR tests for causal estimation of RA on AD, two of which address horizontal pleiotropy, and we performed additional sensitivity analyses. All Mendelian Randomization results were consistent with lack of association between RA and AD (OR = 1.018, 95% CI 0.98–1.06 for IVW, OR = 1.04, 95% CI 0.98–1.11 for Median Weighted OR = 1.05, 95%CI 0.94–1.17 for MR-Egger). We also found no evidence for unbalanced pleiotropy in the genetic instrument as indicated by the P-value of the MR-Egger intercept (p = 0.51), that provides additional confidence in the MR results.

### Possible explanations for inconsistency between observational and MR findings

This was the first meta-analysis to investigate the effect on AD development of RA itself (irrespective of NSAID use) since McGeer *et al*. in 1996^22^, and the only one to produce both separate and an overall OR from case control and population-based studies. A more recent meta-analyses included studies that included dementia (and not only AD) as well as results from non peer-reviewed journals^42^. Attempts were made to ensure lack of publication bias and an influence analysis was carried out to ensure that no one study was predominantly affected the results. Study year, study type, study quality, participant age, RA incidence in controls, and type of arthritis investigated were shown to have no significant effect on the results. Publication bias was also shown to be insignificant.

### Heterogeneity and small number of studies included

However, this meta-analysis also had limitations; owing to the limitations inherent to observational studies, heterogeneity was high due to differences in study design and population, despite taking factors that could have influenced it into consideration. Additionally, there were a small number of studies included in the meta-analyses and no eligible studies after 2002. The low number of studies that met the inclusion criteria meant that all were included, regardless of their quality rating using the NOS. Ideally, only those of medium or high quality would be included. However, including the quality of the study as a covariate did not influence the results of the meta-analysis. Additionally, the majority of the included studies were case control and utilized small study samples; however, the results of the only two longitudinal population-based studies included also showed a relationship between RA and AD. Ideally, more population-based studies, which confer advantages such as larger sample size, a more representative sample and the ability to estimate the prevalence rate of risk factors in the target population, would be carried out and included in a meta-analysis on this topic. In population-based studies, cofounders to exposures and outcomes can also be evaluated, reducing bias^43^.

### RA incidence and diagnosis

Another possible limitation of the meta-analysis was the large variation in RA incidence in controls given across the studies included in the meta-analysis analysis, ranging from 3.8–68%. While this could be due to a number of factors, including differing sources of controls, different sample sizes and different methods of diagnosing RA, it is worth considering as a factor that may have affected the results. In all but one of the studies (Jenkinson *et al*.^27^), the presence of RA was confirmed using structured interview or questionnaire. Ideally, it would be formally diagnosed by a clinician. Additionally, in studies that may have included osteoarthritis (“arthritis” was investigated), incidence in both cases and controls was higher than those that investigated RA alone, as expected. However, the variation of RA proportion in controls was not found to have a significant effect on the results. Additionally, incidence in cases correlated highly with that in controls (rho = 0.947, p = 1.0 * 10^−4^).

### NSAID medication

The links between RA and AD have previously been described as due to a protective effect of NSAIDs. Both RA/arthritis and NSAID use were investigated as risk factors for AD in several studies included in this analysis. Most notably, Lindsay *et al*. found that both arthritis and NSAID use were protective when used in the same model alongside age, sex and education. Similar odds ratios (0.61 for arthritis and 0.65 for NSAID use) were generated for both of them, and their interaction was found to be non-significant (p = 0.10)^29^. Importantly, year of study may affect the treatment taken by included RA patients. Pharmacological therapies for RA have evolved over the past 25 years, with biologic response modifier treatments such as tumor necrosis factor alpha (TNF-α) inhibitors becoming available in the late 1990s^44^ – after most of the studies in this review were carried out. The antifolate drug methotrexate has become the initial treatment in many RA patients, and can be used as a monotherapy without the addition of NSAIDs or any other drugs^44^. The wide variety of RA treatments available today means that participants of earlier studies were probably more likely to be taking NSAIDs to treat their disease, while participants in later studies were possibly taking newer drugs (although NSAIDs are still used in RA treatment). This is particular relevant as this meta-analysis also suffered from lack of recent data, with only three included studies having been carried out since the year 2000. Additionally, of the studies that did not investigate NSAID use and arthritis separately, there is no way to confirm that the RA patients involved were not taking NSAIDs, meaning that this meta-analysis did not investigate RA incidence completely independent of NSAID use.

### Exclusion of relevant studies

Finally, we also excluded a number of studies that did not match our criteria. A 1994 study by Myllykangas-Luosujarvi and Isomaki^45^ investigated the number of AD deaths in RA patients, compared to the entire Finnish population of age 55+. They found that 1.2% of RA patients (2/167) were diagnosed with AD at death, compared to 5.4% (227/4204) of the general population. A 1990 study by McGeer *et al*.^33^ was also excluded; this study found that out of 923 patients in RA clinic, only 4 (0.4%) had AD, while 2/409 (0.5%) patients at AD clinic had RA. They compared this to the incidence of AD in the general population, considered in this study to be 2.7%. Recently, a large population study by Bauer *et al*.^46^ compared comorbidity including arthritis in 9,139 elderly German individuals with dementia and 28,614 German age- and gender-matched control subjects aged 65 years and older. They reported that the presence of arthritis was less frequent in dementia patients (OR = 0.82, 95% CI 0.76–0.88, p < 0.0001) although this association was less strong when only community-living individuals were examined (OR = 0.93, 95% CI 0.85–1.01, p = 0.102). However, this study was also excluded from our meta-analyses as it included patients with other types of dementia in addition to AD, such as vascular dementia. Similarly, a more recent study investigating the association between several chronic inflammatory disorders, including RA and anti-inflammatory drugs, and all-cause dementia was also excluded from our meta-analysis^47^. This study showed that RA, amongst other inflammatory disorders, was associated with higher dementia risk only in treated patients, suggesting a modifiable role of drug therapy in the association between inflammation and dementia risk and it also reported that combined glucocorticoid and NSAID anti-inflammatory therapy was associated with lower risk of dementia among individuals with rheumatoid arthritis.

Finally, a recent study by Wallin *et al*.^48^ was the only population study to show an increased risk for AD and dementia amongst RA patients. However, this study was not included in our meta-analysis as it differed slightly from the others in that patients with mild cognitive impairment (MCI) were included in addition to AD patients and ordinal regression analysis results were reported^48^ instead of an OR of AD risk in RA compared to controls. When we repeated our meta-analysis including the ordinal regression results by Wallin *et al*.^48^, we observed no association between RA and AD for the population-based cohorts (OR = 1, 95% CI 0.45–2.20). However there was no overall difference to the overall meta-analysis result (OR = 0.67, 95%CI 0.48–0.93). With the exception therefore of the study by Wallin *et al*.^48^ these population-based studies also support the finding of the meta-analysis, although an overall OR comparable to that of the other studies was not produced due to variations in outcomes investigated.

### MR strengths and limitations

The MR study has a number of obvious strengths. MR studies minimize bias inherent to observational studies such as confounding, regression dilution bias and reverse causation. However, MR studies are susceptible to bias from pleiotropy (association of genetic variants with more than one variable). Although vertical pleiotropy does not necessarily breach the assumptions of MR, horizontal pleiotropy could invalidate the MR assumption of the genetic variant only affecting the outcome conditional on the exposure of interest, and potentially lead to biased causal estimates. Although the inclusion of multiple variants in MR analysis typically leads to increased statistical power it also results in the potential inclusion of pleiotropic genetic variants that are not valid IVs. To minimize horizontal pleiotropy we employed a weighted median estimator, which provides valid estimates even if 50% of the SNPs are not valid instruments and we employed MR-Egger regression to provide a test for unbalanced pleiotropy and a causal estimate of exposure on outcome in its presence. Our results using all three approaches were consistent, and the MR-Egger approach showed no evidence for unbalanced pleiotropy as indicated by intercept P-value. Even if MR-Egger results in loss of precision and power, our weighted median estimator results were also very similar to the IVW estimator providing additional confidence for these associations.

Another possible limitation to this MR study is the strength of our IV. Although we used 62 established RA SNPs, a score including more RA associated SNPs would have more power to detect a causal effect. To this end, we also repeated our analyses including SNPs associated with RA below genome-wide significance (total of 90 SNPs) but results were almost identical. Also, larger RA studies and meta-analyses are necessary in order to obtain stronger estimates with narrower confidence intervals. Finally, a number of additional analyses can be performed. For example, genetic variants can be categorized as relating to different disease mechanisms and separate MR estimates can be obtained using each category of variants^37^. For example, variants may be associated with RA by various mechanisms, such as chronic inflammation or immune dysregulation. A MR estimate constructed using variants associated with RA through chronic inflammation more closely represents the causal effect of intervening on AD via inflammatory processes. Differences in the causal estimates using genetic variants associated with different mechanisms may be informative in understanding the aetiology of the disease, and may highlight specific mechanisms to prioritize for pharmacological intervention^37^.

## Conclusion

Our study shows that although there seems to be an inverse association between incidence of RA and AD based on observational studies, MR analysis points to lack of a causal association. Examining the results from different methods that make different assumptions (IVW, Weighted Median, MR-Egger regression) provides a sensitivity analysis that either adds to or questions the robustness of a finding from a MR investigation. Here, the same result is reported across all methods, making the findings more plausible than if the methods gave contradictory findings. The protective associations therefore observed in the observational studies could be due to bias inherent to observational studies such differential RA diagnosis, type of medication, small number of small size studies and selection bias. Further, well designed epidemiological studies and MR studies utilizing larger number of instruments and individuals could help draw more confident conclusions about the association of the two diseases.

## Methods

### Systematic review

#### Systematic review search strategy

This review was carried out according to the Preferred Reporting Items for Systematic Reviews and Meta-Analyses (PRISMA) Guidelines^49^.

A search in October 2016 in a) MEDLINE and b) the Cochrane Library was carried out using the following search terms:

1. Alzheimer*, dementia
2. Rheumatoid arthritis, risk factor
3. Incidence, prevalence, epidemiolog*

Combination of these terms (1 ∩ 2 ∩ 3), resulted in 7587 publications. Initial screening, which searched for publications relevant to the aims of this study, resulted in 16 publications being retained for evaluation. Their references were then searched for further relevant publications. Further screening according to the inclusion and exclusion criteria of this review (described below) resulted in 10 studies being included.

#### Inclusion Criteria

In order to be included in this review, studies were selected on the basis of their:

1. Publication in English
2. Publication in a peer-reviewed journal
3. Full text availability
4. Description of empirical research methods relevant to the aims of this review
5. Comparison of the variable studied against a control group
6. Investigation using RA as risk factor for AD
7. The use of clinically diagnosed AD patients. Studies investigating other forms of dementia such as mild cognitive impairment (MCI) and vascular dementia were not included

The search process is summarised in Supplementary Figure 1.

#### Methodological Quality Assessment of Included Studies

Methodological quality assessment was carried out on each of the 12 included studies.

Studies were assessed using the Newcastle-Ottawa Quality Assessment Scale (NOS). Using this scale, studies are assessed and awarded stars based on three domains: selection of study groups (maximum 4 stars), comparability of study groups (maximum 2 stars), and ascertainment of exposure (maximum 3 stars). Slightly different versions of the scale are used for case control and population-based studies^50^.

A study with a score of 5 or less was considered of low quality, a score of 6–7 was considered medium quality, and a score or 8 or 9, high quality. The quality results are shown in Additional File 1. The use of the NOS resulted in 2 studies being deemed high quality, 6, of medium quality and 2 of low quality.

#### Meta-Analysis of Studies

Random-effects meta-analysis was performed using the “metan” command in STATA 12 (StataCorp, College Station, TX, USA). Subgroup analysis was undertaken first, based on the type of study (case-control or population-based study), and then the results of these were combined to produce an overall odds ratio (OR).

Heterogeneity of studies was assessed using Cochran’s Q statistic to calculate Higgins and Thompson’s I^2^, a measure of the proportion of the total variability that can be explained by study heterogeneity. This was assessed first for each group of studies (case control and population-based), and then overall.

To estimate potential bias, the influence of individual studies on the summary effect estimate was first investigated using the “metainf” command. This performs an influence analysis, in which the meta-analysis estimates are computed omitting one study at a time.

A funnel plot was used to visualize the variation of each study (using the “metafunel” command) followed by the Begg’s and Egger’s tests to investigate for funnel-plot asymmetry in meta-analysis (using the “metabias” command). If individual studies were found to have a large influence or introduce potential bias, analysis was repeated omitting them and results were compared.

Finally, the “metareg” command was used to perform meta-regression analysis to investigate the associations between the outcome of the study (AD or control) and study characteristics that may result in study heterogeneity such as study year, study type (case control or population-based study), study quality, age of study participants, incidence of RA in controls and whether arthritis or RA specifically was investigated.

8 case control and 2 population-based studies were included in the meta-analysis.

### Mendelian Randomization (MR)

#### Inverse-Variance Weighted (IVW) instrumental variable analyses

To investigate the causal associations between RA and AD we used summary statistics from the largest published RA Genome-wide Association (GWA) study and meta-analysis to date^51^ and summary data from the International Genomics of Alzheimer’s Project (IGAP)^52^, the largest GWA study and meta-analysis of AD reported to date. Of the 102 SNPs (101 loci) reported to be associated with RA by Okada *et al*.^51^, we excluded 2 SNPs located on the X chromosome as there were no available IGAP data^2^, 2 SNPs that were not directly genotyped or imputed, 7 SNPs that were not directly genotyped or imputed and which were additionally not associated with RA in individuals of European ancestry at genome-wide significance (p < 5 * 10^−8^) in combined meta-analyses and 1 SNP that was not directly genotyped or imputed and for which there was no available summary data in Okada *et al*. for individuals of European ancestry in combined meta-analyses. Of the 90 SNPs (89 loci) that were directly genotyped or imputed, 28 SNPs were further excluded as they were not associated with RA at genome-wide significance (p < 5 * 10^−8^). We therefore used 62 SNPs (61 loci) that were directly genotyped or imputed and which were associated with RA in individuals of European ancestry at genome-wide significance (p < 5 * 10^−8^) in combined meta-analyses (Supplementary Table 2). We extracted the 62 summary SNP associations with AD from IGAP’s stage 1 results (17,008 AD cases and 37,154 controls) and selected as the “risk” allele that which was associated with increased RA risk.

The instrumental variable (IV) estimate from summary data was then obtained by summing the log Odds Ratio (log OR) of the individual logistic regression analyses of all 61 SNPs against AD and weighing this with the summary of the estimates of each SNP obtained from Okada *et al*.^51^ in an IVW meta-analysis (conventional MR) applying the Johnson formula described by Burgess *et al*.^53^. The delta method was used to approximate the standard error. We used only the first-order term from the delta expansion here; further terms were not considered because they did not affect estimates or standard errors.

Although the inclusion of multiple variants in a MR analysis typically leads to increased statistical power, it also presents challenges such as the potential inclusion of pleiotropic genetic variants that are not valid IVs. Whereas vertical pleiotropy does not necessarily breach the assumptions of MR, horizontal pleiotropy can lead to spurious conclusions about causality rendering the IVW estimator inefficient, especially when the precision of the individual estimates varies considerably.

#### MR-Egger instrumental variable analyses

To account for unmeasured horizontal pleiotropy, we performed MR-Egger analysis, an alternative summary data analysis method^39^ that tests for presence of, and accounts for, unbalanced pleiotropy by introducing a parameter for this bias. MR-Egger tests the hypothesis that the strength of the IV estimates of individual SNPs is symmetrically distributed around the point estimate. Symmetrical distribution suggests that pleiotropic effects, if present, are balanced and should not systematically bias the estimate of causal effect. Briefly, MR-Egger performs a weighted linear regression of the gene-outcome coefficients on the gene-exposure coefficients. The slope of this regression represents the causal effect estimate and the intercept can be interpreted as an estimate of the average (horizontal) pleiotropic effect across the genetic variants. MR-Egger regression replaces the second and third IV assumptions with the InSIDE assumption that states that the individual SNP effects on the exposure are independent of their pleiotropic effects on the outcome^39^.

#### Weighted Median Estimator instrumental variable analyses

We additionally used the weighted median estimator, a method using summary data that offers protection against invalid instruments and provides a consistent estimate of causal effect if at least 50% of the weight comes from valid IVs^40^. We assume that no single IV contributes more than 50% of the weight, otherwise the 50% validity assumption is equivalent to assuming that this IV is valid. Analogously to the IVW method, we adopted the inverse of the variance of the ratio estimates as weights, as previously suggested^40^. The weighted median estimator has the advantage of retaining greater precision in the estimates compared to MR-Egger^40^.

#### Sensitivity analyses

A number of sensitivity analyses were finally performed to examine the stability of the summary causal estimate. Firstly, we performed a “leave one out” analysis to further investigate the possibility that the causal association was driven by a single SNP. We further we examined the stability of the summary causal estimate by repeatedly (100,000 times) excluding ~20% SNPs (12 SNPs), with replacement, from the instrument chosen at random in each cycle and collecting the resulting IV estimates. When more than 5% of the sensitivity coefficients lay outside the CI from the normal distribution of the estimate with complete data, there was evidence that the result was sensitive to SNP selection.

All MR analyses were performed in R 3.3.1.

### Data availability

We have used publicly available data for this work.

## Electronic supplementary material

Supplementary Material
