# Supplementary material for: Rheumatoid arthritis and risk for Alzheimer’s disease: a systematic review and meta-analysis and a Mendelian Randomization study
Source: Sci Rep. 2017 Oct 9;7:12861. doi: 10.1038/s41598-017-13168-8 (PMC5634412; doi:10.1038/s41598-017-13168-8)
Supplement: Supplementary file 1 — Supplementary Material [file 41598_2017_13168_MOESM1_ESM.pdf]

# Rheumatoid arthritis and risk for Alzheimer's disease: a systematic review and meta-analysis and a Mendelian Randomization study

**Stefania Policicchio[1,2+], Aminah Noor Ahmad[3+], John Francis Powell[1$], and Petroula Proitsi[1$]***

[1]King's College London, Institute of Psychiatry, Psychology and Neuroscience, London, UK
[2]University of Exeter Medical School, Royal Devon & Exeter NHS Foundation Trust, RILD Medical Research-Complex Disease Epigenetics Group, Exeter, UK
[3]King's College London, School of Medical Education, London, UK
*petroula.proitsi@kcl.ac.uk
[+] These authors contributed equally to this work
[$] These authors contributed equally to this work

**Supplementary Figure 1. A diagrammatic representation of the literature review search process.**
The search process involved finding suitable studies to evaluate, beginning with the initial search, which yielded 4520 results. Further screening for relevance and by study design reduced this number, with 10 studies eventually being included for review.

**Supplementary Figure 2**. Funnel plot evaluating variation of individual studies as a function of their OR and their variance.

**Supplementary Table 1. The results of the NOS Scale, used on the studies included in this review.**

**Supplementary Table 2. SNPs used for the Mendelian Randomization analyses.**
Only SNPs associated with RA at Genome–wide significance in the European Population (combined stages 1+2+3) were used. A1 is the RA risk SNP.

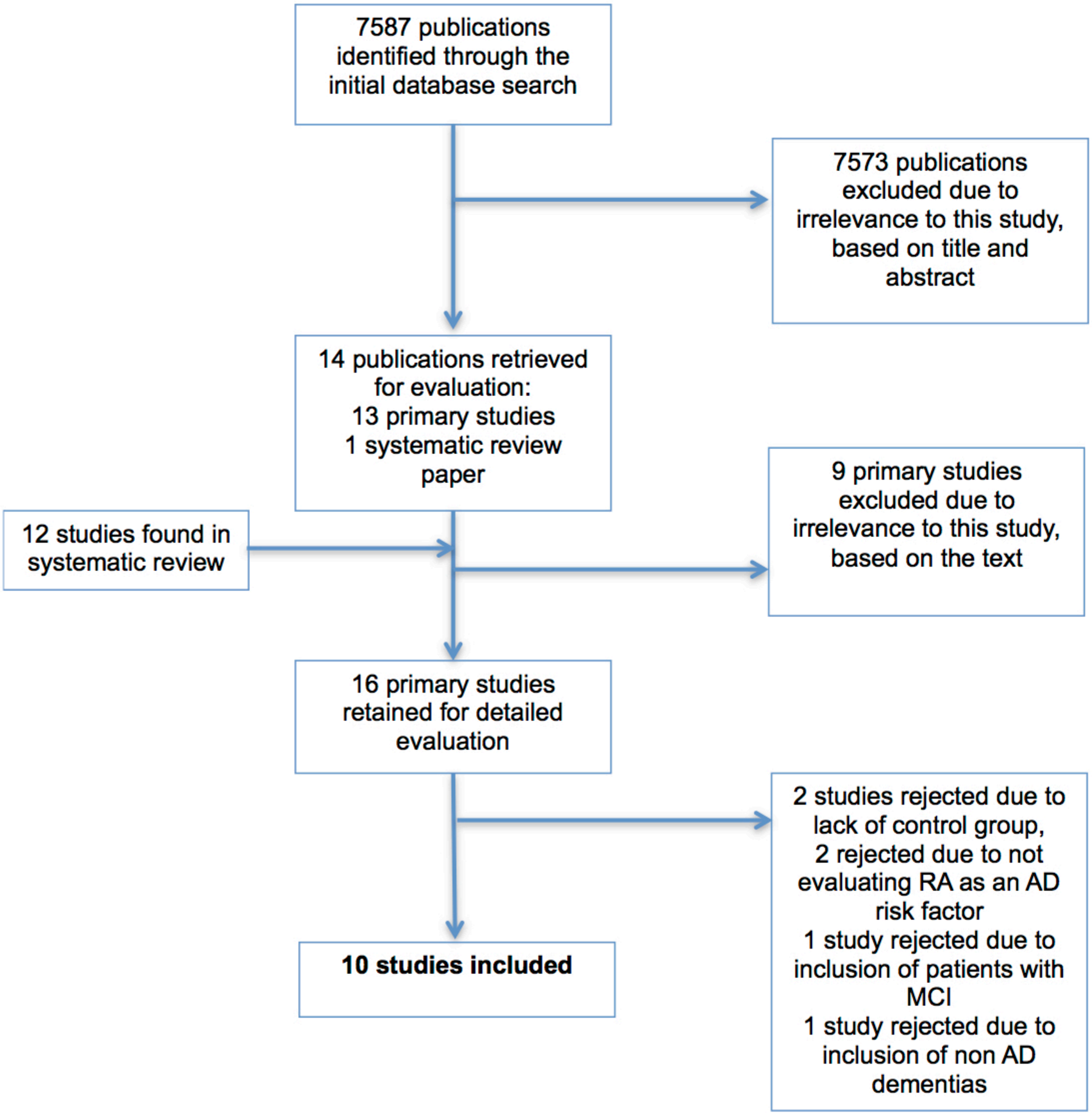
7587 publications identified through the initial database search
7573 publications excluded due to irrelevance to this study, based on title and abstract
14 publications retrieved for evaluation: 13 primary studies 1 systematic review paper
12 studies found in systematic review
9 primary studies excluded due to irrelevance to this study, based on the text
16 primary studies retained for detailed evaluation
2 studies rejected due to lack of control group, 2 rejected due to not evaluating RA as an AD risk factor 1 study rejected due to inclusion of patients with MCI 1 study rejected due to inclusion of non AD dementias
10 studies included

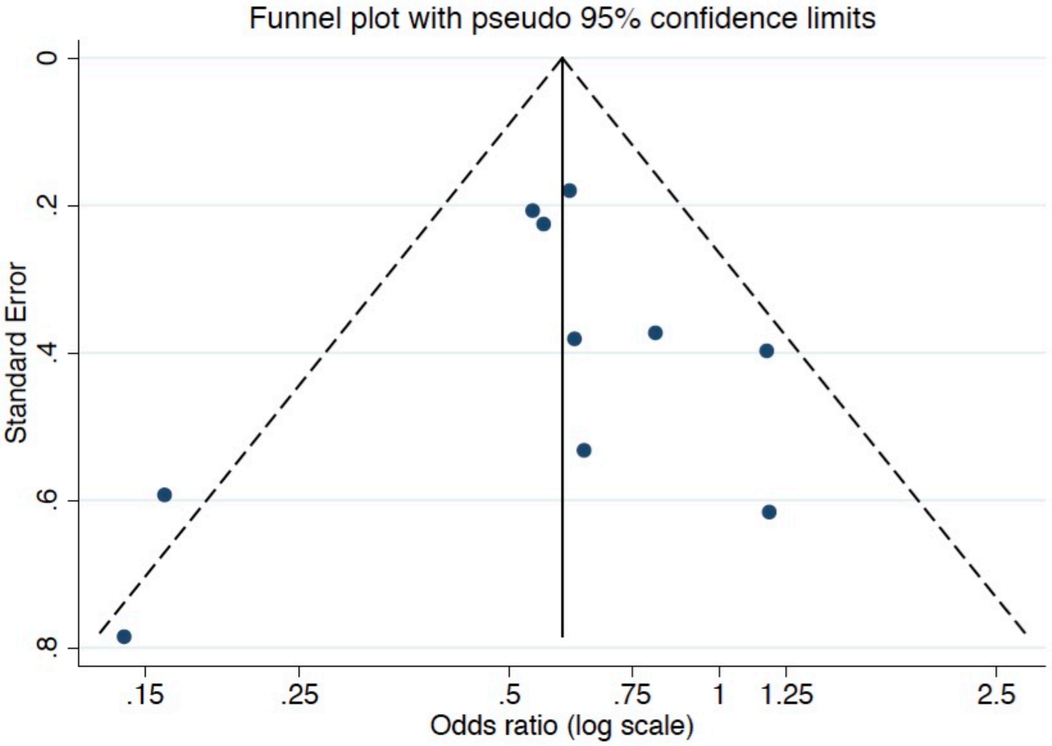

Funnel plot with pseudo 95% confidence limits
Standard Error
0
.2
.4
.6
.8
.15
.25
.5
.75
1
1.25
2.5
Odds ratio (log scale)

| Study | Selection | Comparability | Outcome | Total |
|---|---|---|---|---|
| **Case Control Studies** | | | | |
| Heyman et al **[31]** | ★★★ | ★★ | ★ | 6/9 (medium) |
| French et al **[23]** | ★★★ | ★★ | ★★ | 7/9 (medium) |
| Jenkinson et al **[24]** | ★★ | ★ | ★★ | 5/9 (low) |
| Graves et al **[32]** | ★★★ | ★★ | ★ | 6/9 (medium) |
| Broe et al **[25]** | ★★★ | ★★ | ★★ | 7/9 (medium) |
| Li et al **[26]** | ★★★ | ★★ | ★ | 6/9 (medium) |
| Can. Health **[27]** | ★★★★ | ★★ | ★ | 7/9 (medium) |
| Brietner et al **[28]** | ★★ | ★★ | ★ | 5/9 (low) |
| **Population-Based Studies** | | | | |
| Tyas et al **[29]** | ★★★★ | ★★ | ★★★ | 9/9 (high) |
| Lindsay et al **[30]** | ★★★★ | ★★ | ★★★ | 9/9 (high) |

| SNP | Chr | Position (bp) | Gene | A1 | A2 | OR (95% CI) | Pvalue | SNPs used | Reason for exclusion |
|---|---|---|---|---|---|---|---|---|---|
| chr1:2523811 | 1 | 2,523,811 | TNFRSF14-MMEL1 | G | A | 1.10 (1.07-1.14) | 4.80E-09 | NA | Not genotyped/imputed |
| rs227163 | 1 | 7,961,206 | TNFRSF9 | C | T | 1.00 (0.97-1.03) | 9.30E-01 | NA | Below GWA threshold |
| rs2301888 | 1 | 17,672,730 | PADI4 | G | A | 1.11 (1.07-1.14) | 5.50E-09 | rs2301888 | NA |
| rs28411352 | 1 | 38,278,579 | MTF1-INPP5B | T | C | 1.10 (1.07-1.14) | 5.90E-09 | rs28411352 | NA |
| rs12140275 | 1 | 38,633,879 | LOC339442 | A | T | 1.11 (1.07-1.14) | 4.40E-09 | rs12140275 | NA |
| rs2476601 | 1 | 114,377,568 | PTPN22 | A | G | 1.80 (1.73-1.88) | 8.90E-170 | rs2476601 | NA |
| rs624988 | 1 | 117,263,790 | CD2 | T | C | 1.09 (1.06-1.12) | 8.00E-10 | rs624988 | NA |
| rs2228145 | 1 | 154,426,970 | IL6R | A | C | 1.07 (1.04-1.10) | 4.80E-06 | NA | Below GWA threshold |
| rs2317230 | 1 | 157,674,997 | FCRL3 | T | G | 1.06 (1.03-1.09) | 1.90E-04 | NA | Not genotyped/imputed and below GWA threshold |
| rs4656942 | 1 | 160,831,048 | LY9-CD244 | G | A | 1.01 (0.98-1.05) | 4.80E-01 | NA | Below GWA threshold |
| rs72717009 | 1 | 161,405,053 | FCGR2A | T | C | 1.12 (1.07-1.18) | 5.20E-07 | NA | Below GWA threshold |
| chr1:161644258 | 1 | 161,644,258 | FCGR2B | C | G | - | - | NA | Not genotyped/imputed and no P-value |
| rs2105325 | 1 | 173,349,725 | LOC100506023 | C | A | 1.12 (1.08-1.15) | 3.30E-11 | rs2105325 | NA |
| rs17668708 | 1 | 198,640,488 | PTPRC | C | T | 1.12 (1.06-1.18) | 1.80E-05 | NA | Below GWA threshold |
| rs10175798 | 2 | 30,449,594 | LBH | A | G | 1.09 (1.06-1.12) | 4.20E-08 | NA | Not genotyped/imputed |
| rs34695944 | 2 | 61,124,850 | REL | C | T | 1.13 (1.09-1.16) | 3.70E-16 | rs34695944 | NA |
| rs13385025 | 2 | 62,461,120 | B3GNT2 | A | G | 1.08 (1.02-1.15) | 1.00E-02 | NA | Below GWA threshold |
| rs1858037 | 2 | 65,598,300 | SPRED2 | T | A | 1.09 (1.06-1.13) | 2.00E-09 | rs1858037 | NA |
| rs9653442 | 2 | 100,825,367 | AFF3 | C | T | 1.12 (1.09-1.15) | 9.80E-15 | rs9653442 | NA |
| rs6732565 | 2 | 111,607,832 | ACOXL | A | G | 1.10 (1.07-1.14) | 9.40E-09 | rs6732565 | NA |
| rs11889341 | 2 | 191,943,742 | STAT4 | T | C | 1.12 (1.09-1.16) | 1.40E-12 | rs11889341 | NA |
| rs6715284 | 2 | 202,154,397 | CFLAR-CASP8 | G | C | 1.15 (1.10-1.20) | 2.50E-09 | rs6715284 | NA |
| rs1980422 | 2 | 204,610,396 | CD28 | C | T | 1.13 (1.09-1.17) | 1.90E-13 | rs1980422 | NA |
| rs3087243 | 2 | 204,738,919 | CTLA4 | G | A | 1.15 (1.12-1.18) | 3.60E-22 | rs3087243 | NA |
| rs4452313 | 3 | 17,047,032 | PLCL2 | T | A | 1.11 (1.08-1.15) | 5.20E-11 | rs4452313 | NA |
| rs3806624 | 3 | 27,764,623 | EOMES | G | A | 1.08 (1.05-1.12) | 2.80E-08 | rs3806624 | NA |
| rs73081554 | 3 | 58,302,935 | DNASE1L3-ABHD6-PXK | T | C | 1.18 (1.11-1.25) | 4.70E-08 | rs73081554 | NA |
| rs9826828 | 3 | 136,402,060 | IL20RB | A | G | 1.44 (1.28-1.61) | 8.70E-10 | rs9826828 | NA |
| rs13142500 | 4 | 10,727,357 | CLNK | C | T | 1.10 (1.06-1.15) | 2.40E-06 | NA | Below GWA threshold |
| rs11933540 | 4 | 26,120,001 | C4orf52 | C | T | 1.15 (1.11-1.19) | 9.50E-17 | rs11933540 | NA |
| rs2664035 | 4 | 48,220,839 | TEC | A | G | 1.08 (1.05-1.11) | 3.30E-08 | rs2664035 | NA |
| rs10028001 | 4 | 79,502,972 | ANXA3 | T | A | 1.02 (0.98-1.06) | 2.70E-01 | NA | Below GWA threshold |
| rs45475795 | 4 | 123,399,491 | IL2-IL21 | G | A | 1.14 (1.08-1.20) | 3.50E-06 | NA | Below GWA threshold |
| rs7731626 | 5 | 55,444,683 | ANKRD55 | G | A | 1.21 (1.17-1.26) | 8.20E-23 | rs7731626 | NA |
| rs2561477 | 5 | 102,608,924 | C5orf30 | G | A | 1.11 (1.08-1.14) | 2.20E-11 | rs2561477 | NA |
| rs657075 | 5 | 131,430,118 | IL3-CSF2 | A | G | 1.07 (1.01-1.12) | 1.10E-02 | NA | Below GWA threshold |

| | | | | | | | | | |
|---|---|---|---|---|---|---|---|---|---|
| rs9378815 | 6 | 426,155 | IRF4 | C | G | 1.09 (1.05-1.12) | 1.40E-07 | NA | Not genotyped/imputed and below GWA threshold |
| chr6:14103212 | 6 | 14,103,212 | CD83 | T | C | 1.10 (1.02-1.18) | 1.30E-02 | NA | Below GWA threshold |
| rs9268839 | 6 | 32,428,772 | HLA-DRB1 | G | A | 2.47 (2.39-2.55) | <1.0E250 | rs116633882 | NA |
| rs2234067 | 6 | 36,355,654 | ETV7 | C | A | 1.14 (1.09-1.19) | 4.10E-08 | rs2234067 | NA |
| rs2233424 | 6 | 44,233,921 | NFKBIE | T | C | 1.33 (1.20-1.47) | 3.30E-08 | rs2233424 | NA |
| rs9372120 | 6 | 106,667,535 | ATG5 | G | T | 1.10 (1.06-1.14) | 3.80E-08 | rs9372120 | NA |
| rs17264332 | 6 | 138,005,515 | TNFAIP3 | G | A | 1.17 (1.13-1.21) | 4.10E-20 | rs17264332 | NA |
| rs7752903 | 6 | 138,227,364 | TNFAIP3 | G | T | 1.41 (1.31-1.52) | 1.70E-20 | rs7752903 | NA |
| rs9373594 | 6 | 149,834,574 | PPIL4 | T | C | 1.07 (1.02-1.12) | 6.50E-03 | NA | Not genotyped/imputed and below GWA threshold |
| rs2451258 | 6 | 159,506,600 | TAGAP | T | C | 1.10 (1.07-1.13) | 1.60E-10 | rs2451258 | NA |
| rs1571878 | 6 | 167,540,842 | CCR6 | C | T | 1.13 (1.10-1.17) | 2.40E-18 | rs1571878 | NA |
| rs67250450 | 7 | 28,174,986 | JAZF1 | T | C | 1.11 (1.07-1.14) | 2.60E-09 | rs67250450 | NA |
| rs4272 | 7 | 92,236,829 | CDK6 | G | A | 1.10 (1.07-1.14) | 1.20E-08 | rs4272 | NA |
| chr7:128580042 | 7 | 128,580,042 | IRF5 | G | A | 1.12 (1.08-1.15) | 4.10E-12 | rs3778753 | NA |
| rs2736337 | 8 | 11,341,880 | BLK | C | T | 1.09 (1.06-1.13) | 7.60E-08 | NA | Below GWA threshold |
| rs998731 | 8 | 81,095,395 | TPD52 | T | C | 1.09 (1.06-1.12) | 6.60E-09 | rs998731 | NA |
| rs678347 | 8 | 102,463,602 | GRHL2 | G | A | 1.10 (1.06-1.13) | 7.30E-09 | rs678347 | NA |
| rs1516971 | 8 | 129,542,100 | PVT1 | T | C | 1.16 (1.11-1.21) | 3.20E-11 | rs1516971 | NA |
| rs11574914 | 9 | 34,710,338 | CCL19-CCL21 | A | G | 1.13 (1.09-1.16) | 1.80E-15 | rs11574914 | NA |
| rs10985070 | 9 | 123,636,121 | TRAF1-C5 | C | A | 1.09 (1.06-1.12) | 4.20E-09 | rs10985070 | NA |
| rs706778 | 10 | 6,098,949 | IL2RA | T | C | 1.12 (1.09-1.15) | 4.60E-15 | rs706778 | NA |
| rs947474 | 10 | 6,390,450 | PRKCQ | A | G | 1.12 (1.08-1.16) | 3.30E-10 | rs947474 | NA |
| rs3824660 | 10 | 8,104,722 | GATA3 | C | T | 1.10 (1.06-1.13) | 2.70E-09 | rs3824660 | NA |
| rs12413578 | 10 | 9,049,253 | 10p14 | C | T | 1.20 (1.12-1.29) | 7.50E-08 | NA | Below GWA threshold |
| rs793108 | 10 | 31,415,106 | ZNF438 | T | C | 1.07 (1.04-1.10) | 6.10E-07 | NA | Below GWA threshold |
| rs2671692 | 10 | 50,097,819 | WDFY4 | A | G | 1.06 (1.03-1.09) | 2.60E-05 | NA | Below GWA threshold |
| rs71508903 | 10 | 63,779,871 | ARID5B | T | C | 1.15 (1.11-1.20) | 3.30E-15 | rs71508903 | NA |
| rs6479800 | 10 | 64,036,881 | RTKN2 | C | G | 1.08 (1.04-1.13) | 1.40E-04 | NA | Below GWA threshold |
| rs726288 | 10 | 81,706,973 | SFTPD | T | C | 0.96 (0.86-1.06) | 4.10E-01 | NA | Below GWA threshold |
| rs331463 | 11 | 36,501,787 | TRAF6-RAG1/2 | T | A | 1.12 (1.07-1.16) | 1.10E-07 | NA | Below GWA threshold |
| rs508970 | 11 | 60,906,450 | CD5 | A | G | 1.07 (1.04-1.10) | 2.70E-06 | NA | Below GWA threshold |
| rs968567 | 11 | 61,595,564 | FADS1-FADS2-FADS3 | C | T | 1.12 (1.07-1.16) | 1.80E-08 | rs968567 | NA |
| rs11605042 | 11 | 72,411,664 | ARAP1 | G | A | 1.05 (1.01-1.09) | 1.40E-02 | NA | Below GWA threshold |
| rs4409785 | 11 | 95,311,422 | CEP57 | C | T | 1.12 (1.08-1.16) | 3.60E-09 | rs4409785 | NA |
| chr11:10796735( | 11 | 107,967,350 | ATM | A | G | 1.21 (1.13-1.29) | 1.10E-08 | rs138193887 | NA |
| rs10790268 | 11 | 118,729,391 | CXCR5 | G | A | 1.17 (1.13-1.22) | 1.40E-17 | rs10790268 | NA |
| rs73013527 | 11 | 128,496,952 | ETS1 | C | T | 1.08 (1.05-1.11) | 1.00E-06 | NA | Below GWA threshold |

| | | | | | | | | | |
|---|---|---|---|---|---|---|---|---|---|
| rs773125 | 12 | 56,394,954 | CDK2 | A | G | 1.09 (1.06-1.12) | 2.10E-08 | rs773125 | NA |
| rs1633360 | 12 | 58,108,052 | CDK4 | T | C | 1.08 (1.05-1.11) | 7.10E-08 | NA | Below GWA threshold |
| rs10774624 | 12 | 111,833,788 | SH2B3-PTPN11 | G | A | 1.09 (1.06-1.13) | 6.90E-09 | rs10774624 | NA |
| rs9603616 | 13 | 40,368,069 | COG6 | C | T | 1.11 (1.07-1.14) | 2.80E-11 | rs9603616 | NA |
| rs3783782 | 14 | 61,940,675 | PRKCH | A | G | 1.12 (0.96-1.31) | 1.40E-01 | NA | Not genotyped/imputed and below GWA threshold |
| rs1950897 | 14 | 68,760,141 | RAD51B | T | C | 1.09 (1.06-1.12) | 5.00E-08 | NA | Below GWA threshold |
| rs2582532 | 14 | 105,392,837 | PLD4-AHNAK2 | C | T | 0.93 (0.72-1.21) | 5.90E-01 | NA | Not genotyped/imputed and below GWA threshold |
| rs8032939 | 15 | 38,834,033 | RASGRP1 | C | T | 1.13 (1.09-1.17) | 3.20E-14 | rs8032939 | NA |
| rs8026898 | 15 | 69,991,417 | LOC145837 | A | G | 1.15 (1.11-1.18) | 5.90E-18 | rs8026898 | NA |
| rs4780401 | 16 | 11,839,326 | TXNDC11 | T | G | 1.09 (1.06-1.13) | 8.70E-09 | rs4780401 | NA |
| rs13330176 | 16 | 86,019,087 | IRF8 | A | T | 1.12 (1.08-1.17) | 9.00E-09 | rs13330176 | NA |
| rs72634030 | 17 | 5,272,580 | C1QBP | A | C | 1.12 (1.06-1.19) | 2.90E-05 | NA | Below GWA threshold |
| rs1877030 | 17 | 37,740,161 | MED1 | C | T | 1.09 (1.05-1.13) | 1.30E-05 | NA | Below GWA threshold |
| chr17:38031857 | 17 | 38,031,857 | IKZF3-CSF3 | G | T | 1.09 (1.06-1.12) | 6.00E-10 | rs59716545 | NA |
| rs8083786 | 18 | 12,881,361 | PTPN2 | G | A | 1.12 (1.08-1.16) | 8.40E-09 | rs8083786 | NA |
| rs2469434 | 18 | 67,544,046 | CD226 | C | T | 1.05 (1.02-1.08) | 6.70E-04 | NA | Below GWA threshold |
| rs34536443 | 19 | 10,463,118 | TYK2 | G | C | 1.46 (1.33-1.60) | 4.60E-16 | rs34536443 | NA |
| chr19:10771941 | 19 | 10,771,941 | ILF3 | C | T | 1.47 (1.30-1.67) | 8.80E-10 | rs147622113 | NA |
| rs4239702 | 20 | 44,749,251 | CD40 | C | T | 1.14 (1.11-1.18) | 1.10E-16 | rs4239702 | NA |
| rs73194058 | 21 | 34,764,288 | IFNGR2 | C | A | 1.13 (1.08-1.18) | 2.60E-08 | rs73194058 | NA |
| chr21:35928240 | 21 | 35,928,240 | RCAN1 | C | T | 1.12 (1.07-1.17) | 7.10E-07 | NA | Not genotyped/imputed and below GWA threshold |
| rs8133843 | 21 | 36,738,242 | RUNX1-LOC100506403 | A | G | 1.09 (1.06-1.12) | 2.00E-08 | rs8133843 | NA |
| rs1893592 | 21 | 43,855,067 | UBASH3A | A | C | 1.11 (1.07-1.15) | 9.80E-09 | rs1893592 | NA |
| rs2236668 | 21 | 45,650,009 | ICOSLG-AIRE | C | T | 1.07 (1.03-1.10) | 4.60E-05 | NA | Not genotyped/imputed and below GWA threshold |
| rs11089637 | 22 | 21,979,096 | UBE2L3-YDJC | C | T | 1.10 (1.06-1.15) | 2.00E-07 | NA | Below GWA threshold |
| rs3218251 | 22 | 37,545,505 | IL2RB | A | T | 1.08 (1.04-1.11) | 5.70E-06 | NA | Below GWA threshold |
| rs909685 | 22 | 39,747,671 | SYNGR1 | A | T | 1.11 (1.08-1.15) | 6.40E-12 | rs909685 | NA |
| chrX:78464616 | X | 78,464,616 | P2RY10 | A | C | 1.16 (0.78-1.75) | 4.60E-01 | NA | Chromosome X data not available |
| rs5987194 | X | 153,301,467 | IRAK1 | C | G | 1.16 (1.12-1.21) | 2.80E-12 | NA | Chromosome X data not available |
